# Supplementary material for: Free Energy Perturbation Calculations of Mutation Effects on SARS-CoV-2 RBD::ACE2 Binding Affinity
Source: J Mol Biol. 2023 Aug 1;435(15):168187. doi: 10.1016/j.jmb.2023.168187 (PMC10286572; doi:10.1016/j.jmb.2023.168187)
Supplement: Supplementary data 1 [file mmc1.pdf]

Supplementary Information for

Free energy perturbation calculations of mutation effects on SARS-CoV-2 RBD::ACE2 binding affinity

Alina P. Sergeeva, Phinikoula S. Katsamba, Junzhuo Liao, Jared M. Sampson, Fabiana Bahna, Seetha Mannepalli, Nicholas C. Morano, Lawrence Shapiro, Richard A. Friesner, Barry Honig

Corresponding authors –
Barry Honig bh6@columbia.edu
Richard A. Friesner raf8@columbia.edu
Lawrence Shapiro lss8@columbia.edu

This PDF file includes:

Figures S1 to S3
Tables S1 to S5

A

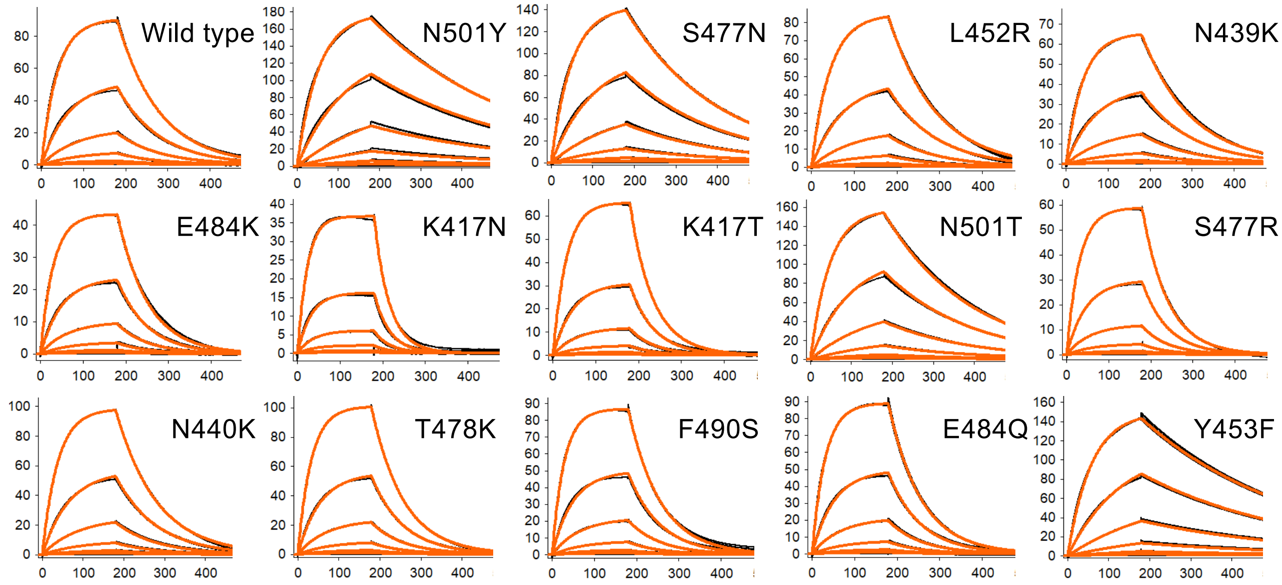

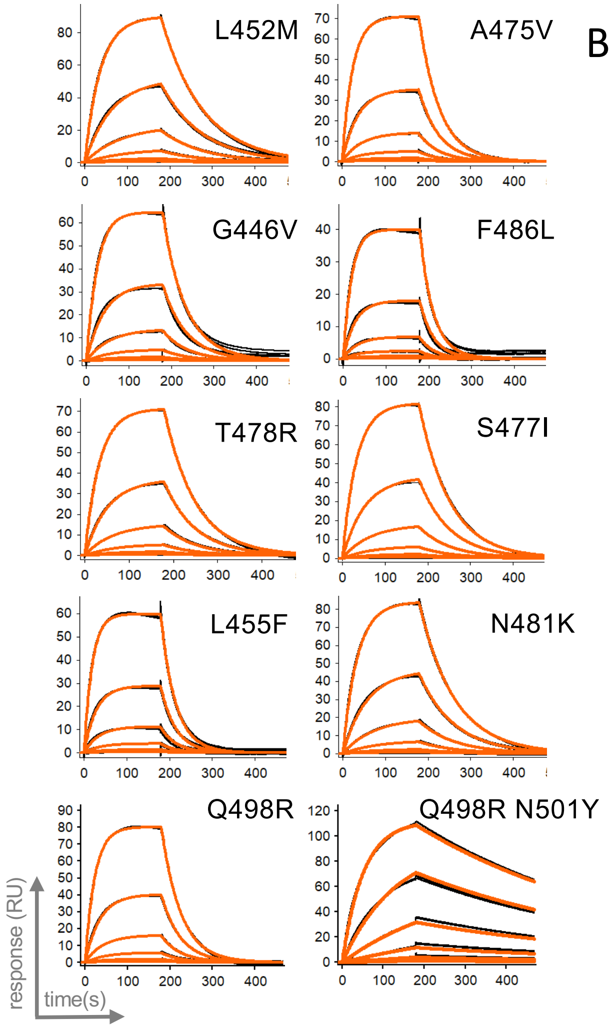

B

RBD mutant	k_a ($M^{-1}s^{-1}$)	k_d (s^{-1})	K_D (nM)
RBD wild type	$6.18x10^4$	$1.01x10^{-2}$	162.9
RBD N501Y	$6.26x10^4$	$2.85x10^{-3}$	45.6
RBD S477N	$6.05x10^4$	$4.54x10^{-3}$	75.2
RBD L452R	$5.20x10^4$	$8.97x10^{-3}$	172.5
RBD N439K	$6.20x10^4$	$8.50x10^{-3}$	137.0
RBD E484K	$7.11x10^4$	$1.40x10^{-2}$	196.4
RBD K417N	$8.54x10^4$	$3.98x10^{-2}$	466.0
RBD K417T	$7.21x10^4$	$2.46x10^{-2}$	342.0
RBD N501T	$6.23x10^4$	$4.73x10^{-3}$	76.0
RBD S477R	$6.31x10^4$	$1.63x10^{-2}$	257.7
RBD N440K	$6.23x10^4$	$9.93x10^{-3}$	159.3
RBD T478K	$6.83x10^4$	$1.28x10^{-2}$	187.6
RBD F490S	$8.25x10^4$	$1.33x10^{-2}$	160.5
RBD E484Q	$7.30x10^4$	$1.31x10^{-2}$	179.3
RBD Y453F	$5.54x10^4$	$2.71x10^{-3}$	48.9
RBD L452M	$6.40x10^4$	$1.03x10^{-2}$	161.3
RBD A475V	$7.67x10^4$	$2.09x10^{-2}$	273.0
RBD G446V	$1.05x10^5$	$2.44x10^{-2}$	233.6
RBD F486L	$1.15x10^5$	$4.98x10^{-2}$	430.0
RBD T478R	$6.08x10^4$	$1.42x10^{-2}$	234.2
RBD S477I	$6.20x10^4$	$1.37x10^{-2}$	221.8
RBD L455F	$1.13x10^5$	$3.56x10^{-2}$	315.7
RBD N481K	$6.50x10^4$	$1.22x10^{-2}$	187.6
RBD Q498R	$1.02x10^5$	$2.20x10^{-2}$	217.2
Q498R N501Y	$8.15x10^4$	$1.84x10^{-3}$	22.6

Figure S1: SPR analysis of wt and mutant RBD/ACE2 binding affinities and kinetic parameters. (A) SPR binding profiles for ACE2 binding to RBD and its mutants. The black traces represent experimental data, overlaid with the red traces which represent the 1:1 interaction model used to extrapolate kinetic parameters. (B) Kinetic parameters determined from the fits shown in A. The error of the fit for k_a is (0.003-0.05)x10^4 $M^{-1}s^{-1}$, for k_d is (0.004-0.6)x10^{-3} s^{-1} and for K_D is 0.07-7 nM.

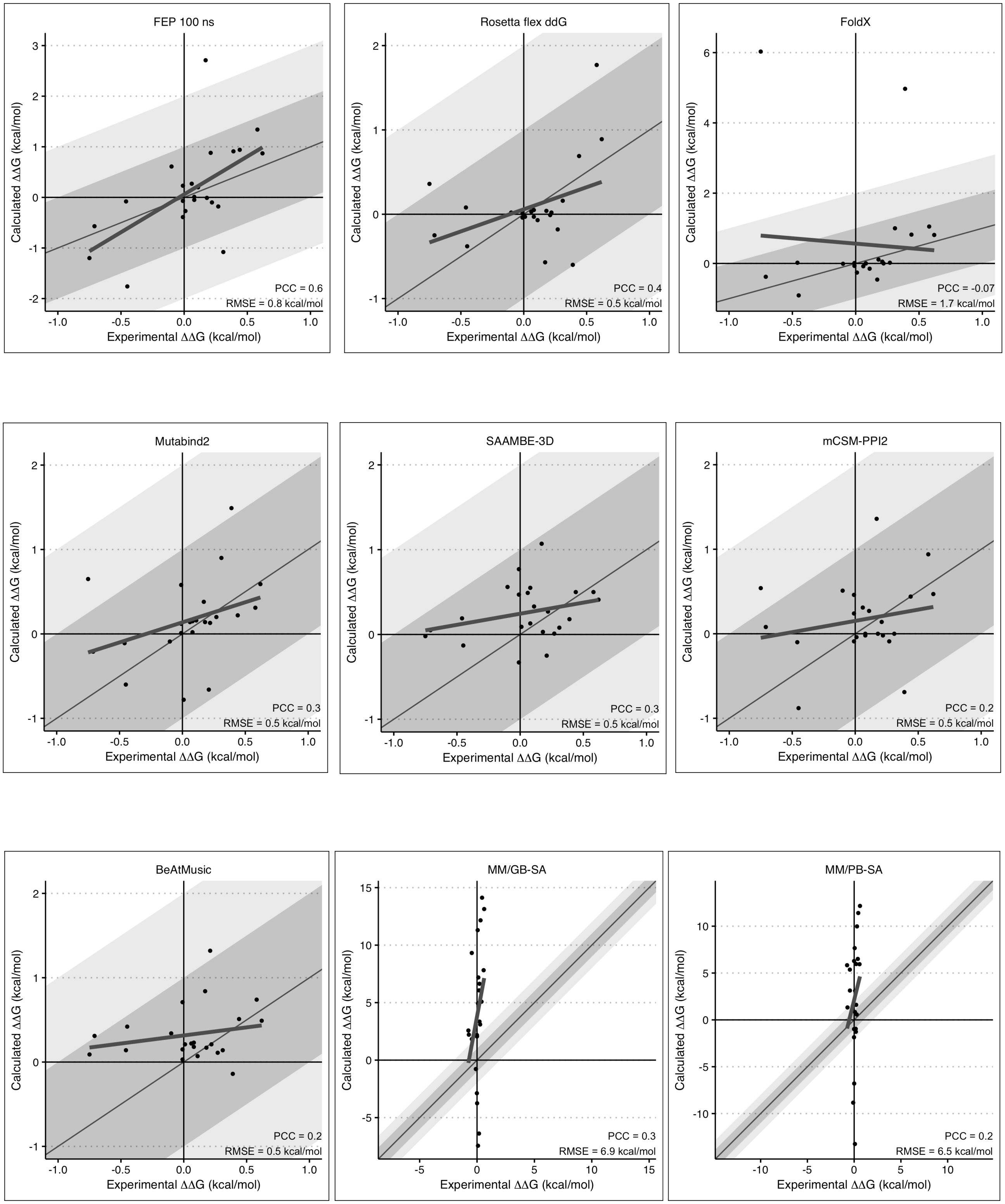

Figure S2. Correlation plots for data in Table 2.

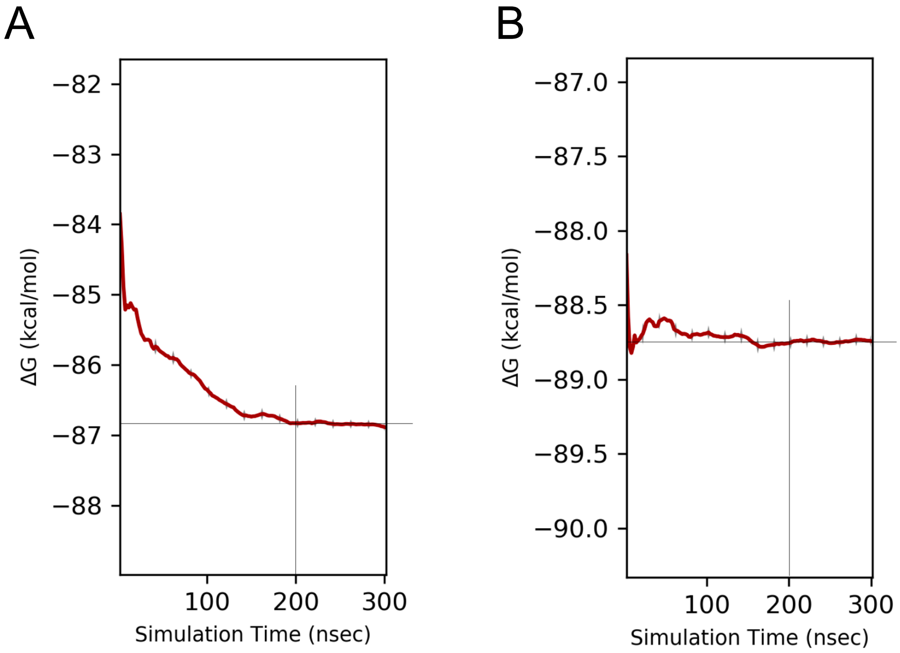

Figure S3. Energy convergence plots for Q498R mutation. Convergence plots of the FEP calculations for the complex leg (A) and the solvent leg (B) of the Q498R mutation simulated for 300ns to demonstrate that energy convergence is achieved at 200ns. ΔΔG (mutation) = ΔG (complex leg) – ΔG (solvent leg); ΔG = G(mutant) - G(wild-type).

Table S1. Occurrence frequency of single point SARS-CoV-2 RBD mutations in the US in 2021.

RBD mutation	Total frequency, Jan 04'21	Total frequency, Jan 20'21	Total frequency, Feb 19'21	Total frequency, Mar 10'21	Total frequency, April 18'21	Total frequency, May 24'21	Total frequency, June 7'21	Total frequency, July 26'21	Total frequency, Aug 31'21	Total frequency, Oct 31'21	Total frequency, Dec 12'21	Total frequency, Dec 31'21
L452R	213	465	1141	1247	10056	25430	77577	180087	492276	1094585	1663119	1875810
T478K	13	35	177	190	4948	14146	62325	161860	467835	1069045	1637282	1849188
N501Y	373	469	11873	12081	169174	388294	614750	711234	778190	822805	837129	840571
E484K	82	192	487	592	9432	31142	60178	77492	97264	116482	122751	124058
K417T	0	0	17	40	1508	8481	23113	33295	47315	62212	65836	66643
S477N	12833	14448	16695	17160	22109	26139	29491	31840	33170	34071	34939	35592
N439K	3971	5435	6625	6872	10027	11899	14295	15617	16505	18474	19124	19388
K417N	19	21	90	102	1653	3993	6461	7733	9415	11179	11855	12188
E484Q	19	21	34	35	201	1062	2682	3102	3624	4953	7010	7926
F490S	25	27	37	42	388	1633	2740	3155	5971	6350	6572	6702
G446V	36	47	57	58	122	182	376	693	1272	2819	4179	4997
N440K	32	46	80	84	464	892	1741	2987	4091	4485	4847	4995
S477I	112	119	135	135	186	286	401	543	1012	1968	2982	3381
N501T	87	232	451	479	1382	1672	1880	2013	2265	2346	2429	2527
A475V	27	50	65	67	123	185	559	875	1382	1475	1560	1609
L455F	32	35	36	36	131	304	462	574	772	1063	1283	1373
S477R	144	250	321	329	538	740	812	839	847	883	907	924
L452M	54	62	66	67	137	382	632	713	791	854	874	896
Y453F	161	163	177	178	185	232	241	261	276	286	310	330
T478R	13	17	33	37	79	90	105	150	171	236	280	303
Q498R	0	0	0	0	0	0	0	5	12	35	71	207
F486L	90	90	138	138	139	142	144	148	150	153	155	156
N481K	13	37	47	47	65	98	95	103	109	129	134	134

Table S2. RBD mutations present in select SARS-CoV-2 variants.

Variant, WHO classification	Importance	PANGO Lineage	Country first detected	RBD Mutations
Alpha (α)	VOC	B.1.1.7	United Kingdom	N501Y
Beta (β)	VOC	B.1.351	South Africa	K417N, E484K, N501Y
Gamma (γ)	VOC	P.1	Brazil	K417T, E484K, N501Y
Delta (δ)	VOC	B.1.617.2	India	L452R, T478K
Epsilon (ε)	VOI->VUM->reclassified	B.1.427/B.1.429	USA	L452R
Zeta (ζ)	VOI->VUM->reclassified	P.2	Brazil	E484K
Eta (η)	VOI->VUM	B.1.525	Nigeria	E484K
Theta (θ)	VOI->VUM->reclassified	P.3	The Philippines	E484K, N501Y
Iota (ι)	VOI->VUM	B.1.526	USA	S477N, E484K
Kappa (κ)	VOI->VUM	B.1.617.1	India	L452R, E484Q
Lambda (λ)	VOI	C.37	Peru	L452Q, F490S
Mu (μ)	VOI	B.1.621	Colombia	R346K, E484K, N501Y
Omicron (ο)	VOC	B.1.1.529	South Africa	G339D, S371L, S373P, S375F, K417N, N440K, G446S, S477N, T478K, E484A, Q493R, G496S, Q498R, N501Y, Y505H
"Stealth" omicron	VOC	BA.2	South Africa	G339D, S371F, S373P, S375F, T376A, D405N, R408S, K417N, N440K, S477N, T478K, E484A, Q493R, Q498R, N501Y, Y505H
-	-	B.1.258	Scotland	N439K
-	-	A.28	France	E484K, N501T
-	-	B.1.1.298 (cluster 5)	Denmark/Netherlands (mink)	Y453F

Single point mutations experimentally probed in the current study are highlighted in blue.

Importance

VOC	variant of concern
VOI	variant of interest
VUM	variant under monitoring
reclassified	Former VOCs/VOIs/VUMs, including their descendent lineages, that have been reclassified based on at least one the following criteria:<br>(1) the variant is no longer circulating at levels of global public health significance,<br>(2) the variant has been circulating for a long time without any impact on the overall epidemiological situation, or<br>(3) scientific evidence demonstrates that the variant is not associated with any concerning properties.

Table S3. Comparison of FEP to experimental SPR values and other theoretical methods. Color code and notations as in Table 2. The length of FEP simulation slightly affects performance. Longer (100ns) FEP runs help with convergence and show slightly better performance compared to 10ns simulations.

Complex	Mutation	ΔΔG experiment SPR	ΔΔG FEP 10ns	ΔΔG FEP 100ns	ΔΔG Mutabind2	ΔΔG mCSM-PPI2	ΔΔG SAAMBE-3D	ΔΔG BeAtMusic	ΔΔG FoldX	ΔΔG Rosetta flex ddG	ΔΔG MM/PB-SA	ΔΔG MM/GB-SA
ACE2::RBD	N501Y	-0.8	0.6	-1.2	0.7	0.5	0.0	0.1	6.0	0.4	5.8	2.6
ACE2::RBD	Y453F	-0.7	-0.9	-0.6	-0.2	0.1	0.1	0.3	-0.4	-0.2	1.3	2.2
ACE2::RBD	S477N	-0.5	-0.4	-0.1	-0.1	-0.1	0.2	0.1	0.0	0.1	5.4	9.3
ACE2::RBD	N501T	-0.5	-2.1	-1.8	-0.6	-0.9	-0.1	0.4	-0.9	-0.4	3.1	1.8
ACE2::RBD	N439K	-0.1	-0.3	0.6	-0.1	0.5	0.6	0.3	0.0	0.0	-8.8	-0.8
ACE2::RBD	N440K	0.0	-0.2	-0.4	0.1	-0.1	0.5	0.0	-0.1	0.0	-1.9	-2.9
ACE2::RBD	F490S	0.0	-0.4	-0.1	0.6	0.2	0.8	0.7	0.0	0.0	-1.0	2.2
ACE2::RBD	L452M	0.0	0.5	0.2	0.0	0.5	-0.3	0.2	0.0	0.0	6.3	2.1
ACE2::RBD	L452R	0.0	-0.2	-0.3	-0.8	0.0	0.1	0.2	-0.3	0.0	-6.8	-3.8
ACE2::RBD	E484Q	0.1	0.7	0.3	0.1	0.3	0.5	0.2	-0.1	0.0	7.7	11.3
ACE2::RBD	T478K	0.1	0.0	0.0	0.2	0.0	0.1	0.2	0.0	0.1	0.8	4.9
ACE2::RBD	N481K	0.1	0.1	-0.1	0.0	0.0	0.6	0.2	0.0	0.0	-13.2	-7.4
ACE2::RBD	E484K	0.1	0.5	0.2	0.2	0.3	0.3	0.1	-0.2	-0.1	3.1	7.2
ACE2::RBD	Q498R	0.2	3.6	2.7	0.4	1.4	1.1	0.8	-0.5	-0.6	-1.0	-6.4
ACE2::RBD	S477I	0.2	-0.2	0.0	0.1	0.0	0.0	0.2	0.1	0.0	-1.3	6.1
ACE2::RBD	G446V	0.2	-0.1	0.4	-0.7	0.1	-0.3	1.3	0.1	0.0	6.0	6.6
ACE2::RBD	T478R	0.2	0.3	-0.1	0.1	0.0	0.3	0.2	0.0	0.0	1.6	3.4
ACE2::RBD	S477R	0.3	-0.3	-0.2	0.2	-0.1	0.0	0.1	0.0	-0.2	0.6	3.1
ACE2::RBD	A475V	0.3	-0.7	-1.1	0.9	0.0	0.1	0.1	1.0	0.2	10.0	12.2
ACE2::RBD	L455F	0.4	2.2	1.7	1.5	-0.7	0.2	-0.1	5.0	-0.6	6.5	5.1
ACE2::RBD	K417T	0.4	0.9	0.9	0.2	0.4	0.5	0.5	0.8	0.7	11.4	14.1
ACE2::RBD	F486L	0.6	1.2	1.3	0.3	0.9	0.5	0.7	1.1	1.8	5.9	7.8
ACE2::RBD	K417N	0.6	1.3	0.9	0.6	0.5	0.4	0.5	0.8	0.9	12.2	13.1
Dpr10/DIP-α	Dpr10 Q142M	-1.3	-0.2	-0.3	-0.1	0.3	0.4	-0.2	0.0	0.2	-3.6	-1.6
Dpr10/DIP-α	DIP-α A78K	-1.1	-1.0	-1.0	0.5	-0.1	-1.4	1.2	-0.5	-0.1	1.5	5.6
Dpr6/DIP-α	DIP-α A78K	-1.0	-0.9	-1.0	0.8	-0.1	-1.3	1.3	-0.3	-0.2	3.2	-2.5
Dpr6/DIP-α	DIP-α G74A	-0.5	0.9	0.7	-1.0	0.1	0.8	0.1	-0.3	0.8	3.0	-1.1
Dpr6/DIP-α	DIP-α N94D	-0.5	0.4	0.4	1.4	0.0	0.9	0.3	0.1	0.1	10.6	1.4
Dpr10/DIP-α	DIP-α G74A	-0.4	1.4	1.2	-0.1	0.3	0.7	-0.1	-0.3	0.8	-5.2	-0.6
Dpr10/DIP-α	DIP-α N94D	-0.1	0.4	0.0	1.1	0.0	0.5	0.2	0.1	0.3	-6.2	-3.0
Dpr6/DIP-α	DIP-α A82T	0.9	2.2	2.1	1.1	-0.5	0.2	0.6	1.9	1.1	6.0	0.5
Dpr10/DIP-α	Dpr10 V144K	1.3	3.4	2.8	0.5	0.1	0.3	1.4	0.5	0.6	-5.7	-0.5
Dpr6/DIP-α	DIP-α K81Q	1.3	1.9	1.8	0.9	0.9	0.6	0.9	1.1	-0.3	-1.3	-4.7
Dpr10/DIP-α	DIP-α A82T	1.3	2.2	2.1	0.9	-0.6	0.0	0.4	0.9	0.3	0.4	3.0
Dpr6/DIP-α	DIP-α G74L	1.4	5.2	3.6	2.2	0.5	0.7	-0.1	6.7	4.6	8.8	3.3
Dpr10/DIP-α	Dpr10 Q138D	1.4	2.4	1.3	2.7	1.6	0.7	1.1	3.8	2.2	14.0	2.6
Dpr10/DIP-α	DIP-α K81Q	1.9	2.3	2.4	0.6	0.7	0.4	0.8	1.5	0.8	-8.0	-2.5
Dpr10/DIP-α	DIP-α G74L	1.9	5.9	4.4	2.3	0.6	0.6	-0.3	6.6	3.4	-1.9	2.9
Dpr6/DIP-α	Dpr6 H110K	1.9	1.6	1.6	1.0	0.4	0.5	0.6	0.5	0.8	15.5	7.2
Dpr10/DIP-α	DIP-α I91A	2.1	3.0	2.9	1.5	0.9	1.1	2.0	1.3	0.9	3.3	4.9
Dpr6/DIP-α	DIP-α I91A	2.2	3.4	3.4	2.0	0.8	1.1	1.8	1.8	1.0	9.1	5.4

**ACE2::RBD dataset**	PCC	0.5	0.6	0.3	0.2	0.3	0.2	-0.1	0.4	0.2	0.3
	RMSE	1.0	0.8	0.5	0.5	0.5	0.5	1.7	0.5	6.5	6.9
**DIP::Dpr dataset**	PCC	0.8	0.9	0.6	0.5	0.5	0.3	0.6	0.4	0.2	0.4
	RMSE	1.7	1.2	1.0	1.1	1.1	1.1	1.9	1.3	6.9	3.1
**Combined dataset**	PCC	0.8	0.8	0.6	0.4	0.5	0.4	0.4	0.5	0.2	0.1
	RMSE	1.3	1.0	0.8	0.8	0.8	0.8	1.8	0.9	6.7	5.6
	MCC (stab)	0.5	0.5	0.2	0.0	0.4	-	0.4	0.1	-0.2	0.1
	MCC (dest)	0.6	0.7	0.5	0.6	0.3	0.6	0.9	0.7	0.1	0.2
	MCC (neutral)	0.6	0.6	0.4	0.4	0.2	0.4	0.6	0.6	-0.1	-
	MCC (triple class)	0.6	0.6	0.4	0.4	0.3	0.4	0.7	0.5	-0.1	0.1
	p-value for PCC	3.5E-09	3.0E-11	1.1E-05	4.8E-03	1.2E-03	6.1E-03	2.1E-03	2.2E-04	1.3E-01	2.3E-01

Table S4. Evaluating the reproducibility of FEP predictions on crystal structures. Color code and notations as in Table 3.

Complex	Mutation	ΔΔG experiment SPR kcal/mol	ΔΔG single 100ns FEP run (reported in Table S3)	Average ΔΔG ± stdev (based on ten independent 100ns FEP runs)	SEM
Dpr6/DIP-α	DIP-α A78K	-1.0	-0.9	-0.9 ± 0.1	0.03
ACE2::RBD	N501Y	-0.8	-1.2	-1.4 ± 0.4	0.12
ACE2::RBD	Y453F	-0.7	-0.6	-0.6 ± 0.1	0.04
Dpr6/DIP-α	DIP-α G74A	-0.5	0.7	0.7 ± 0.1	0.03
ACE2::RBD	S477N	-0.5	-0.1	-0.1 ± 0.1	0.04
ACE2::RBD	N501T	-0.5	-1.8	-1.9 ± 0.2	0.06
ACE2::RBD	G446V	0.2	0.4	0.4 ± 0.4	0.13
ACE2::RBD	A475V	0.3	-1.1	-0.9 ± 0.1	0.04
ACE2::RBD	L455F	0.4	1.7	1.7 ± 0.3	0.08
Dpr6/DIP-α	DIP-α A82T	0.9	2.1	2.1 ± 0.2	0.06
Dpr6/DIP-α	DIP-α K81Q	1.3	1.8	1.9 ± 0.1	0.04
Dpr6/DIP-α	DIP-α G74L	1.4	3.6	3.5 ± 0.2	0.07

Table S5. Comparison of binding affinity changes measured and calculated in the current study to previously reported computational predictions. ΔΔG values are given in units of kcal/mol.

RBD mutation	ΔΔG, experiment, SPR, kcal/mol	ΔΔG FEP+, 100ns	ΔΔG FEP, Fratev	ΔΔG FEP, Huynh	ΔΔG FEP, Gumbart	ΔΔG TopNetTree, Wei	ΔΔG AMBER/MMPBSA, Pricl	ΔΔG PRODIGY/ MMGBSA, Wei	ΔΔG PRODIGY/ MMGBSA, Bahar	ΔΔG PRODIGY/ MMPBSA, Zonta	ΔΔG NN_MMGBSA, Maranas with *	*-mutations NN_MMGBSA was trained on
N501Y	-0.8	-1.2	-1.4	-0.8	-4.5	0.0		0.1	-0.4	0.4	-0.1	N501Y*
S477N	-0.5	-0.1				-0.1					-0.1	S477N (S477D*)
L452R	0.0	-0.3				-2.5					-0.1	L452R (L452K,Q*)
N439K	-0.1	0.6				-1.4					-0.1	N439K*
E484K	0.1	0.2			-1.3	-0.9		-0.3		-0.1	-0.1	E484K (E484R*)
K417N	0.6	0.9	0.6		0.4	-1.8				0.1	0.3	K417N (K417E,R,V*)
K417T	0.4	0.9				-1.1	1.5			0.2	0.3	K417T (K417E,R,V*)
N501T	-0.5	-1.8	-1.9			-1.4	-0.3				-0.1	N501T*
S477R	0.3	-0.2				-0.3						
N440K	0.0	-0.4				-0.4					-0.1	N440K*
T478K	0.1	0.0				-0.2					-0.1	
F490S	0.0	-0.1				-0.6					-0.1	F490S (F490K*)
E484Q	0.1	0.3				1.2					-0.1	E484Q (E484R*)
Y453F	-0.7	-0.6				-0.6					-0.1	Y453F*
L452M	0.0	0.2				-2.4						
A475V	0.3	-1.1				1.1						
G446V	0.2	0.9				0.4						
F486L	0.6	1.3				-0.7						
T478R	0.2	-0.1				0.1						
S477I	0.2	0.0				-0.3						
L455F	0.4	0.9				0.7						
N481K	0.1	-0.1				0.1						
Q498R	0.2	2.7				-0.3						

	PCC	0.6				0.0					0.7	
	RMSE	0.8				1.1					0.3	
